# Age-stratified selection of albumin-based inflammatory ratios: a novel strategy for optimizing risk prediction of severe pneumonia in children

**DOI:** 10.3389/fped.2025.1679957

**Published:** 2025-11-14

**Authors:** Yunbao Bai, Jianming Zhang, Changxu Hou

**Affiliations:** Department of Pediatrics, Fuyang Women and Children's Hospital, Fuyang, China

**Keywords:** community-acquired pneumonia, albumin ratios, age stratification, NPAR, CAR, negative predictive value

## Abstract

**Background:**

This study aimed to optimize risk prediction of severe community-acquired pneumonia (CAP) in children through age-stratified selection of albumin-based inflammatory ratios.

**Methods:**

This retrospective study analyzed 1,071 pediatric CAP patients (aged 1–12 years). Propensity score matching (1:2 severe-to-mild ratio) within three age strata (1–3 years, 4–6 years, 7–12 years) generated balanced cohorts (*N* = 360). Neutrophil percentage-to-albumin ratio (NPAR), C-reactive protein-to-albumin ratio (CAR), and CRP × lymphocyte-to-albumin ratio (CALLY) were evaluated using multivariate logistic regression and ROC analysis.

**Results:**

Severe CAP incidence increased with age (1–3 years: 7.37%; 4–6 years: 12.80%; 7–12 years: 18.04%). Multivariate analysis identified NPAR as the sole independent predictor for younger children (1–3 years: OR = 10.289; 4–6 years: OR = 35.117), while CAR predicted severe CAP in older children (7–12 years: OR = 3.342). ROC analysis demonstrated robust performance for NPAR in 1–6 years (AUC: 0.748–0.807, NPV: 85.9–88.0%) and CAR in 7–12 years (AUC: 0.734, NPV: 83.0%).

**Conclusions:**

NPAR (for ages 1–6 years) and CAR (for ages 7–12 years) serve as effective, age-specific biomarkers for severe CAP, facilitating precise risk stratification with high negative predictive value.

## Introduction

Community-acquired pneumonia (CAP) is one of the leading causes of morbidity and mortality among children under 5 years old globally (1). Data from 2016 showed that lower respiratory tract infections caused a total of 2,377,697 deaths worldwide, including 652,572 deaths among children under 5 years old. Sub-Saharan Africa had the highest proportion (27.4%), followed by South Asia (24.8%); the proportion in Latin America and the Caribbean was 6.8% (2–4). In Colombia, the mortality rate of children under 5 years old due to acute respiratory infections reached 14.89 per 100,000 in 2018 (5). Approximately 155 million new cases of CAP occur globally each year (with an incidence of about 0.22 episodes per child per year), of which as many as 10%–17% require hospitalization (4). Patients with CAP present with varying severity levels. Most have mild CAP and can be treated as outpatients with low mortality rates. Moderate CAP patients require hospitalization but generally survive despite higher mortality risks. Severe CAP cases necessitate ICU management and have extremely high short- and long-term mortality rates (6).

Accurate risk stratification of pediatric CAP severity remains challenging due to heterogeneous clinical presentations and the lack of universally validated biomarkers. Current prediction tools [e.g., Pneumonia Severity Index (PSI), CURB-65] (7) exhibit limited generalizability in children owing to developmental variations in immune responses and pathophysiology. These scores were primarily derived and validated in adult populations, incorporating parameters that are developmentally inappropriate or less relevant in pediatric patients (8). Serum albumin (Alb), a negative acute-phase protein reflecting systemic inflammation and nutritional status, has emerged as a promising biomarker substrate (9). However, isolated albumin measurements lack age-specific discriminatory power. Combining albumin with dynamic inflammatory markers [e.g., C-reactive protein (CRP), neutrophils] into ratios such as Neutrophil-Percentage-to-Albumin Ratio (NPAR), CRP to Alb ratio (CAR), and C-reactive protein-albumin-lymphocyte (CALLY) may amplify prognostic precision by integrating dual pathways of inflammation and metabolic stress. Crucially, pediatric immunity evolves across developmental stages: toddlers (1–3 years) exhibit innate immunity dominance with neutrophil-predominant responses, whereas school-aged children (7–12 years) demonstrate adaptive immune maturation with lymphocyte/CRP interplay. This immunophysiological transition likely modulates biomarker performance but is rarely addressed in existing models. We hypothesize that age-stratified selection of albumin-based ratios would optimize severe CAP prediction by aligning biomarker dynamics with host biology. Validate NPAR, CAR, and CALLY as age-specific predictors of severe CAP in three pediatric age strata (1–3 years, 4–6 years, 7–12 years), thereby enabling precision risk stratification for timely intervention.

## Materials and methods

### Study design and participants

This retrospective cohort study analyzed 1,071 pediatric patients (aged 1–12 years) with CAP admitted to Fuyang Women and Children's Hospital between January 2022 to December 2024. Patients were stratified into three developmental stages: 1–3 years (toddlers), 4–6 years (preschoolers), and 7–12 years (school-aged children). Severe CAP was defined as requiring intensive respiratory support (mechanical ventilation/high-flow oxygen) or exhibiting septic shock, consistent with IDSA/ATS minor/major criteria adaptations for pediatrics. Djudication of Severe CAP: The diagnosis of severe CAP was initially established in real-time by the attending pediatric intensivist or emergency physician based on standard clinical criteria. For the purpose of this study, all cases meeting the severe CAP definition were independently reviewed and confirmed by two senior pediatricians who were blinded to the biomarker data. Any discrepancies between the reviewers were resolved through discussion and consensus. Inclusion criteria: Radiologically confirmed CAP (chest x-ray); Availability of complete albumin-based inflammatory ratios (NPAR/CAR/CALLY) within 24 h of admission; Age 1–12 years. Exclusion criteria: Immunosuppression (chemotherapy/HIV); Hospital-acquired pneumonia; Chronic organ failure (renal/liver). Rationale for Age Stratification: Patients were stratified into three age groups (1–3 years, 4–6 years, 7–12 years) based on distinct developmental stages in immunology and respiratory physiology. This approach addresses: (1) Differential immune maturation: Transition from innate immunity dominance (1–3 years) to adaptive immunity competence (7–12 years) (reflected in inflammatory biomarker profiles); (2) Anatomic vulnerability: Smaller airway caliber and weaker cough reflex in toddlers vs. school-aged children.

### Data collection and Variable definitions

Baseline variables were systematically extracted from electronic medical records:

 ① Demographics: Age, sex; ② Clinical parameters: APACHE-II score, renal dysfunction (eGFR <90 ml/min/1.73m^2^), prior pneumonia history; ③ Pathogen data: Viral/bacterial/mixed/other (based on PCR/culture); ④ Inflammatory ratios: NPAR = Neutrophil percentage (%)/Serum albumin (g/dL); CAR = C-reactive protein (mg/L)/Serum albumin (g/dL); CALLY = [CRP (mg/L) × Lymphocyte count (×10^9^/L)]/Serum albumin (g/dL). Lymphocyte and Neutrophil percentage (%) were determined using an XE-2100 hematology analyzer (Sysmex, Kobe, Japan). Serum biochemical parameters, including Albumin (reference range in children: 3.8–5.4 g/dL) and CRP (reference range in children: <5 mg/L), were measured with a HITACHI 7600 automated biochemistry analyzer.

### Statistical analysis

Propensity Score Matching (PSM) was performed separately within each age stratum (1–3 years, 4–6 years, 7–12 years) using the “MatchIt” package (version 4.5.0) in R software. Severe-to-mild matching ratio: 1:2 using nearest-neighbor algorithm (caliper = 0.2 SD); Covariates balanced: Age, sex, APACHE-II, renal dysfunction, pathogen type, pneumonia history (SMD < 0.1); Predictive Modeling: Univariate analysis: Mann–Whitney *U* test (CAR/CALLY)/*t*-test (NPAR) for severe vs. mild CAP; Multivariate logistic regression: Evaluated independent predictors of severe CAP per age stratum; Final model optimization: Retained only age-stratified significant biomarkers (NPAR for 1–6 years, CAR for 7–12 years). Validation: Discriminative power: ROC curves with AUC/95% CI calculation; Hosmer–Lemeshow test; Clinical utility: Sensitivity, specificity, NPV/PPV at optimal cutoffs.

## Results

### Severe community-acquired pneumonia incidence and propensity score matching in pediatric patients: overall and age-stratified analysis

A total of 1,071 pediatric community-acquired pneumonia (CAP) patients aged 1–12 years (951 mild, 120 severe) demonstrated an overall severe CAP incidence of 11.20%, with age-stratified rates progressively increasing from 7.37% (1–3 years) to 12.80% (4–6 years) and 18.04% (7–12 years). Following propensity score matching at a 1:2 severe-to-mild ratio within each age stratum, balanced cohorts were established: 111 cases (74 mild/37 severe) for 1–3 years, 144 cases (96 mild/48 severe) for 4–6 years, and 105 cases (70 mild/35 severe) for 7–12 years. All baseline covariates—including age, sex, renal dysfunction, pathogen type, pneumonia history, and APACHE-II score—achieved inter-group balance (standardized mean difference <0.1; *p* > 0.05) (Table 1).

**Table 1:** Construction of matched cohorts for mild vs. severe pediatric community-acquired pneumonia (CAP).

Parameter	Overall (1–12 years)	1–3 years group	4–6 years group	7–12 years group
A. Pre-matching cohort				
Total cases (*n*)	1,071	502	375	194
Mild group (*n*)	951	465	327	159
Severe group (*n*)	120	37	48	35
Severe CAP incidence (%)	11.20	7.37	12.80	18.04
B. Matching procedure				
Reference group	—	Severe	Severe	Severe
Matching ratio (severe: mild)	—	1:2	1:2	1:2
C. Post-matching cohort				
Severe group (*n*)	—	37	48	35
Mild group (*n*)	—	74	96	70
Matched cohort total (*n*)	—	111	144	105
D. Covariate balance				
Balance achieved	—	Yes	Yes	Yes

Covariates assessed: Age, sex, renal dysfunction, pathogen type (viral/bacterial/mixed/other), prior pneumonia history, APACHE-II score. Balance defined as standardized mean difference (SMD) < 0.1 and *p* > 0.05 for all variables.

Matching methodology: Propensity score matching (1:2) performed separately within each age stratum using severe cases as reference.

Cohort derivation: Matched cohort size = (Matched mild cases) + (Matched severe cases).

### Univariate analysis of inflammatory ratios

All inflammatory ratios exhibited statistically significant differences between severe and mild CAP across age strata (*p* < 0.001). Specifically, CAR demonstrated consistently higher values in severe cases: median values of 0.25 vs. 0.10 in 1–3 years, 0.41 vs. 0.15 in 4–6 years, and 0.75 vs. 0.25 in 7–12 years patients. In contrast, CALLY showed significantly lower median values in severe groups: 11.25 vs. 26.26 (1–3 years), 3.22 vs. 17.81 (4–6 years), and 2.04 vs. 9.60 (7–12 years). Similarly, NPAR was significantly elevated in severe CAP cases across all age groups: mean values of 1.55 ± 0.42 vs. 1.15 ± 0.42 (1–3 years), 1.87 ± 0.40 vs. 1.41 ± 0.36 (4–6 years), and 1.97 ± 0.47 vs. 1.66 ± 0.33 (7–12 years) (Table 2).

**Table 2:** Comparison of inflammatory ratios between severe and mild CAP across age strata.

Index	Age group	Severe group	Mild group	Statistical value	*P*-value
CAR	1–3 years	0.25 (0.13–0.60)	0.10 (0.03–0.26)	*Z* = −3.489	<0.001
	4–6 years	0.41 (0.18–0.94)	0.15 (0.05–0.36)	*Z* = −4.429	<0.001
	7–12 years	0.75 (0.20–1.73)	0.25 (0.06–0.48)	*Z* = −3.915	<0.001
CALLY	1–3 years	11.25 (3.40–28.18)	26.26 (10.65–113.77)	*Z* = −3.464	<0.001
	4–6 years	3.22 (1.51–9.86)	17.81 (6.51–56.99)	*Z* = −5.750	<0.001
	7–12 years	2.04 (0.82–8.63)	9.60 (4.04–56.70)	*Z* = −4.241	<0.001
NPAR	1–3 years	1.55 ± 0.42	1.15 ± 0.42	*t* = 4.704	<0.001
	4–6 years	1.87 ± 0.40	1.41 ± 0.36	*t* = 7.034	<0.001
	7–12 years	1.97 ± 0.47	1.66 ± 0.33	*t* = 3.528	0.001

CAR and CALLY expressed as median (IQR), analyzed by Mann–Whitney *U* test; NPAR expressed as mean ± SD, analyzed by *t*-test. Units: CAR (mg/g·dL^−1^), CALLY (mg·10^9^/L·g^−1^·dL), NPAR (%·g^−1^·dL).

### Multivariate logistic regression analysis

Multivariate logistic regression identified distinct age-stratified predictors of severe CAP: In the 1–3 years group, only NPAR demonstrated statistical significance (OR = 5.612, 95% CI: 1.539–20.469; *p* = 0.009). For 4–6 years patients, NPAR showed dominant predictive value (OR = 23.918, 95% CI: 6.061–94.386; *p* < 0.001), while CAR failed to reach significance (*p* = 0.052). Conversely, in the 7–12 years cohort, CAR emerged as the sole significant predictor (OR = 2.402, 95% CI: 1.052–5.482; *p* = 0.037), with neither CALLY nor NPAR showing significant associations (both *p* > 0.05) (Table 3).

**Table 3:** Multivariate logistic regression for severe CAP prediction by age group.

Age group	Variable	*β*	SE	Wald *χ*^2^	*P*-value	OR (95% CI)
1–3 years	CAR	1.523	0.897	2.88	0.09	4.584 (0.790–26.609)
	CALLY	−0.001	0.002	0.41	0.522	0.999 (0.995–1.002)
	NPAR	1.725	0.66	6.825	0.009	5.612 (1.539–20.469)
4–6 years	CAR	1.013	0.522	3.768	0.052	2.754 (0.990–7.959)
	CALLY	0	0.001	0.119	0.73	1.000 (0.999–1.002)
	NPAR	3.175	0.7	20.545	<0.001	23.918 (6.061–94.386)
7–12 years	CAR	0.876	0.421	4.332	0.037	2.402 (1.052–5.482)
	CALLY	1.168	0.809	2.085	0.149	3.214 (0.659–15.681)
	NPAR	0.002	0.002	0.048	0.826	0.999 (0.995–1.004)

While OR estimates showed wide confidence intervals (particularly in 4–6 years group), the subsequent ROC analysis confirmed robust discriminative performance of the selected biomarkers across age strata.

### Final predictive models with validation

Final age-optimized models demonstrated strong predictive performance: the NPAR model for 1–3 years (OR = 10.289, 95% CI: 3.221–32.971, *p* < 0.001), NPAR model for 4–6 years (OR = 35.117, 95% CI: 9.358–132.226, *p* < 0.001), and CAR model for 7–12 years (OR = 3.342, 95% CI: 1.619–6.896, *p* = 0.001), all with adequate calibration (Hosmer–Lemeshow *p* > 0.05). The incidence of severe CAP progressively increased with age, and optimal biomarkers shifted from NPAR in younger children (1–6 years) to CAR in older children (7–12 years) (Table 4). NPAR-based models (1–6 years) achieved NPV > 83%, while the CAR model (7–12 years) maintained NPV > 83% (see Table 5).

**Table 4:** Final predictive models with calibration validation.

Age group	Predictor	*β*	SE	Wald χ²	*P*-value	OR (95% CI)	HL test (*P*)
1–3 years	NPAR	2.331	0.594	15.391	<0.001	10.289 (3.221–32.971)	0.234
4–6 years	NPAR	3.56	0.676	27.772	<0.001	35.117 (9.358–132.226)	0.784
7–12 years	CAR	1.206	0.37	10.653	0.001	3.342 (1.619–6.896)	0.071

The wide confidence intervals for the odds ratios, particularly for NPAR in the 4–6 years group, are likely attributable to the limited sample size in the propensity score-matched cohorts—a recognized trade-off for achieving improved covariate balance. It is important to note, however, that the subsequent ROC analysis provided independent validation of the strong and robust discriminative performance of this biomarker (AUC = 0.807), supporting its clinical utility despite the imprecision in the OR point estimate.

**Table 5:** ROC curve analysis of final predictive models by age group.

Age group	Predictor	AUC (95% CI)	Sensitivity (95% CI)	Specificity (95% CI)	PPV (95% CI)	NPV (95% CI)	Optimal Cut off
1–3 years	NPAR	0.748 (0.656–0.825)	0.838 (0.680–0.938)	0.595 (0.474–0.707)	0.508 (0.377–0.639)	0.880 (0.757–0.955)	1.26
4–6 years	NPAR	0.807 (0.733–0.868)	0.771 (0.627–0.880)	0.708 (0.607–0.797)	0.569 (0.440–0.692)	0.859 (0.762–0.927)	1.57
7–12 years	CAR	0.734 (0.639–0.815)	0.686 (0.507–0.831)	0.771 (0.656–0.863)	0.600 (0.433–0.751)	0.830 (0.717–0.912)	0.48

### ROC curve analysis

**Figure 1:**
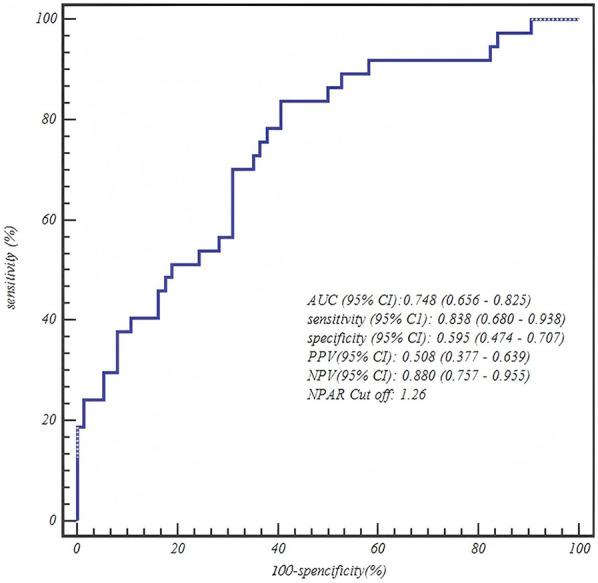
ROC curve of the neutrophil percentage-to-albumin ratio (NPAR) for predicting severe CAP in toddlers (1–3 years).

**Figure 2:**
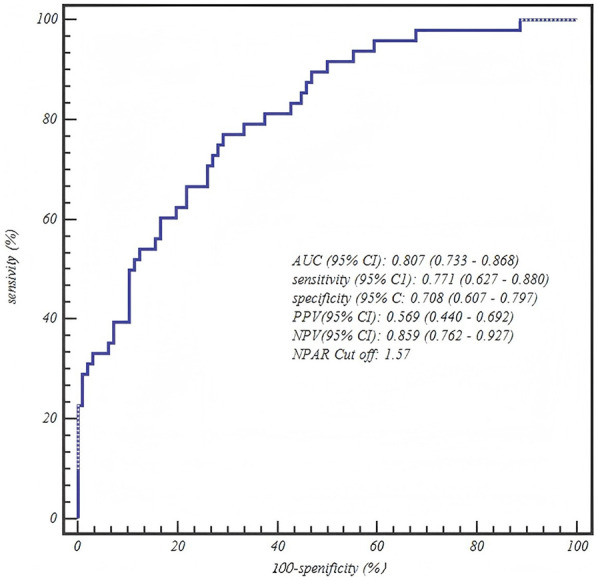
Age-stratified model performance: ROC curve for the neutrophil percentage-to-albumin ratio (NPAR) in predicting severe CAP among children aged 4–6 years.

**Figure 3:**
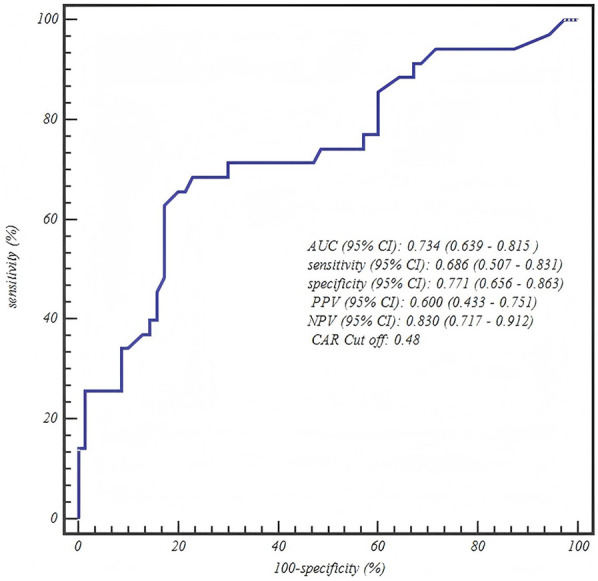
ROC curve of the C-reactive protein-to-albumin ratio (CAR) for predicting severe CAP in school-aged children (7–12 years).

The ROC curve analysis confirmed the robust diagnostic efficacy of our age-stratified models, with all AUCs >0.73 demonstrating clinical utility. Critically, each model achieved high negative predictive value (NPV: 0.83–0.88) for safely excluding severe CAP—particularly vital for 1–3 years toddlers where 88% NPV (0.880, 95% CI: 0.757–0.955) mitigates missed diagnoses. The biologically anchored cutoffs aligned with disease pathophysiology: 1–3 years NPAR > 1.26 (between mild mean 1.15 ± 0.42 and severe mean 1.55 ± 0.42), 4–6 years NPAR > 1.57 (discriminating severe IQR from mild 1.41 ± 0.36), and 7–12 years CAR > 0.48 (2× mild median 0.25). Notably, the 4–6 years NPAR model showed optimal balance (sensitivity 0.771/specificity 0.708, AUC = 0.807), while younger children prioritized sensitivity (0.838) to avert under-triage and adolescents emphasized specificity (0.771) to prevent unnecessary ICU transfers—collectively enabling precision risk-stratification across pediatric developmental stages (Table 5 and Figures 1–3).

## Discussion

CAP is a common and frequently occurring disease of the respiratory system in children. Mild CAP primarily manifests clinically with respiratory symptoms such as fever, cough, and sputum production. Due to fewer alveoli, more developed interstitial tissue, lower lung air volume, and rich pulmonary vasculature in children, inflammation tends to persist and become difficult to resolve. Furthermore, an excessive inflammatory response can cause severe lung damage, making progression to severe CAP more likely (10). When children develop severe CAP, severe hypoxia and toxemia can occur. Beyond symptoms like fever and cough, this can lead to respiratory failure manifestations such as dyspnea and impaired ventilation/perfusion function. It can also cause severe dysfunction in multiple organ systems, including the cardiovascular, nervous, and digestive systems. Severe CAP is a major cause of morbidity and mortality in children worldwide (11). Notably, from a pathophysiological perspective, the systemic inflammatory response and organ dysfunction characterizing severe CAP align with the definition of sepsis, i.e., life-threatening organ dysfunction caused by a dysregulated host response to infection (12). Therefore, it is very important to find early warning indicators and prediction models for severe pneumonia as early as possible.

This study employed rigorous age stratification and propensity score matching (PSM) analysis. Our results revealed a significant age-dependent trend in the incidence of severe CAP among children, which increased from 7.37% in the 1–3 years group to 18.04% in the 7–12 years group. Importantly, baseline characteristics were well-balanced across all age strata after matching. This observed trend is likely explained by age-specific patterns in pathogen distribution and differences in immune system development. In younger children (1–3 years), the risk of severe CAP was relatively low (7.37%). This may be attributed to a virus-dominated infection profile. Supporting this, national surveillance data from Liu et al. indicated a viral detection rate of 50.82% (predominantly RSV and influenza virus) among CAP patients aged ≤5 years, compared to a bacterial positivity rate of only 34.37% (13). Since viral pneumonia is often self-limiting, this pattern aligns with the lower incidence of severe disease we observed in this age group. Conversely, older children (7–12 years) faced a substantially higher risk of severe CAP (18.04%). Two key factors may underlie this increase. First, the pathogen spectrum shifts with age. Liu et al. reported that in children over 6 years, bacterial detection rates (32.53%) become comparable to viral rates (34.37%). Furthermore, viral-bacterial co-infections—such as RSV-Haemophilus influenzae co-infection (OR = 2.62)—were significantly more prevalent in severe CAP cases. Second, immune responses differ in older children. They appear more susceptible to excessive inflammatory reactions. As noted by Principi et al., traditional biomarkers like PCT and CRP can be ambiguous for distinguishing bacterial pneumonia and assessing severity (11). Additionally, the maturation of the immune system with age may sometimes lead to dysregulated inflammatory responses, thereby increasing the risk of tissue damage.

Our findings underscore that the predictive efficacy of albumin-based inflammatory ratios is intrinsically linked to pediatric immune maturation. For toddlers (1–3 years), NPAR emerged as the dominant predictor (OR = 10.289, AUC = 0.748), aligning with their neutrophil-predominant innate immunity. Neutrophils are frontline responders in early childhood infections, and their activity combined with hypoalbuminemia (reflecting acute-phase inflammation) amplifies the signal for severe disease (14). Conversely, in school-aged children (7–12 years), CAR superseded other ratios (OR = 3.342, AUC = 0.734), coinciding with the maturation of adaptive immunity and CRP's role in bacterial challenge amplification (15). This transition mirrors immunological shifts (16, 17): CRP production is more robust in older children due to enhanced hepatocyte response to IL-6, while lymphopenia (integrated in CALLY) may be less specific in bacterial/viral co-infections prevalent in this age group.

Our models' high NPV (83% across all strata) holds transformative potential for clinical practice. In our study on age-stratified albumin-based inflammatory ratios for severe pneumonia risk prediction in children, models like NPAR (1–6 years) and CAR (7–12 years) achieved NPVs of 88.0%, 85.9%, and 83.0% across different age groups. These high NPVs allow safe outpatient management of low-risk cases, reducing unnecessary ICU referrals and optimizing medical resource allocation. This potential is further supported by other clinical studies. In health information exchange (HIE), AI models have demonstrated NPVs of 94.10% to 99.10% in predicting clinical outcomes, as shown in a systematic review (18). Additionally, the ITBvsCD-CEP model achieved an NPV of 83% in differentiating ITB from CD in a multicenter study (19). These high NPVs enhance clinical decision-making, reduce unnecessary interventions, and improve patient care across various medical fields by reliably ruling out conditions. In low-resource settings, NPAR (cutoff >1.26 for 1–3 years; >1.57 for 4–6 years) and CAR (cutoff >0.48 for 7–12 years) can reliably exclude severe CAP, reducing unnecessary ICU referrals. For example, NPV = 88% in toddlers minimizes missed severe cases in a high-volume emergency department. Preschoolers (4–6 years) showed optimal NPAR performance (AUC = 0.807), supporting its use as a first-line tool for this high-risk group.

Our results contrast with adult CAP biomarkers (e.g., PSI/CURB-65), which overlook developmental immunity. While CURB-65 is a validated predictor of mortality from community-acquired pneumonia in adults, it was never intended for use in children (20). Similarly, the PSI and CURB-65 scores have been widely used to predict mortality risk in adult CAP patients, but they may not be as effective in pediatric populations (21). While CAR predicts mortality in adult pneumonia, its age-dependent performance in children—superior only in school-aged cohorts—highlights pediatrics as a distinct biological niche. Similarly, CALLY, validated in chronic inflammation, lacked significance here, possibly due to lymphocyte count variability during acute pediatric infections (22, 23). This reinforces that pediatric biomarker discovery must account for age stratification.

Our definition of severe CAP, based on the requirement for intensive respiratory support or the presence of septic shock, aligns with the modern conceptualization of sepsis (Sepsis-3) (12). Community-acquired pneumonia is one of the most common primary causes of sepsis. Consequently, the most severe form of CAP inherently represents sepsis originating from a pulmonary source. The objective clinical endpoints chosen in this study (e.g., mechanical ventilation and septic shock) accurately capture this critical transition from a localized infection to systemic organ failure. Therefore, the predictive power of NPAR and CAR for severe CAP not only underscores their value in pneumonia severity stratification but also suggests their potential role as early warning biomarkers for identifying children at high risk of progressing from pneumonia to sepsis, providing a stronger conceptual framework for their clinical utility. Weiss et al. validated a multimarker sepsis risk stratification tool in critically ill children, supporting our approach of using inflammatory ratios for early identification of severe CAP progressing to sepsis (24).

The heterogeneity of pediatric CAP, caused by diverse pathogens and host responses, must be considered when interpreting our findings. Although our age-stratified design addressed key immunological differences, the performance of NPAR and CAR within specific subgroups (e.g., viral vs. bacterial infections) requires future investigation. As seen in sepsis research (25), data-driven subphenotyping (e.g., using clustering methods) can reveal distinct subgroups for tailored therapy. Future studies should thus explore how pathogen type and host factors influence these biomarkers to enable truly personalized clinical application. While our study identifies strong associations between NPAR/CAR and severe CAP outcomes, establishing a causal relationship requires further investigation. The observed associations may be influenced by unmeasured confounders or may simply reflect the severity of the underlying inflammatory state. To truly determine if modulating these ratios (e.g., through albumin supplementation or anti-inflammatory therapies) can improve outcomes, future research must move beyond prediction towards causal inference. The framework of target trial emulation (26) provides a rigorous methodology for this purpose. Future prospective studies should be designed to emulate a trial where patients are virtually “randomized” to different management strategies based on their early NPAR/CAR values. This approach would allow us to estimate the causal effect of biomarker-guided therapy on clinical outcomes, ultimately translating these predictive biomarkers into actionable therapeutic targets.

Our study has several limitations. First, as a single-center retrospective analysis, our findings require external validation in diverse populations and healthcare settings to confirm their generalizability, as regional variations in pathogen prevalence and clinical practices may influence biomarker performance. Second, while propensity score matching successfully balanced known confounders, unmeasured covariates (e.g., vaccination status, socioeconomic factors) may still affect the generalizability of our findings. Furthermore, the matching process itself led to a substantial reduction in the analyzable sample size, particularly evident in the 7–12 years cohort (which decreased from 194 to 105 patients). This reduction inevitably diminished the statistical power of our analysis and is a key reason for the wide confidence intervals observed in our multivariate regression models, as noted in the results. Future studies with larger cohorts are needed to obtain more precise estimates. Third, as a retrospective study, we lacked complete data for key variables required to calculate established pneumonia severity scores (e.g., PSI, CURB-65). Consequently, we were unable to directly benchmark the predictive performance of NPAR and CAR against these clinical standards. This important comparison remains a critical objective for future prospective studies that are specifically designed to collect all necessary data for a comprehensive head-to-head evaluation. Fourth, our analysis utilized a single, early measurement of NPAR/CAR (within 24 h of admission) for prediction. While this is clinically relevant for initial triage, it does not capture the temporal dynamics of these biomarkers. As raised The trajectory of NPAR (e.g., its rate of change or persistence at high levels) throughout hospitalization may provide additional prognostic value and reflect response to therapy. Investigating these time-varying patterns represents an important avenue for future research. Finally, the unexpected non-significance of CALLY may reflect lymphocyte count volatility during acute pediatric infections, limiting its utility in CAP risk stratification compared to chronic inflammatory conditions.

For successful translation into clinical practice, the NPAR and CAR ratios can be seamlessly integrated into existing diagnostic workflows. As these ratios are derived from routine laboratory parameters—complete blood count with differentials, CRP, and albumin—they can be automatically calculated and reported alongside standard results by the hospital's laboratory information system or electronic health record. This requires no additional blood draws or costs, presenting a significant advantage for rapid adoption. We propose a straightforward clinical decision pathway: for a child aged 1–6 years with CAP, an NPAR value below the established age-specific cutoff (e.g., >1.26 for 1–3 years; >1.57 for 4–6 years) would support the safety of outpatient management or standard ward care, while a value above the cutoff would flag the need for intensified monitoring and consideration of higher-level care. Similarly, for children aged 7–12 years, the CAR cutoff of >0.48 would guide this triage decision. To definitively establish the value of this biomarker-guided approach, a prospective, multicenter, pragmatic trial is warranted. The critical next step is a head-to-head comparison against current standards of care. This prospective study should be designed to rigorously compare the performance of these age-stratified ratios against both clinical judgment and adapted versions of established adult scores (where feasible in pediatrics) in predicting severe outcomes. The primary goal would be to determine whether the integration of NPAR and CAR into clinical decision rules improves patient outcomes and optimizes resource allocation compared to conventional practices.

## Conclusion

In summary, NPAR and CAR serve as developmentally tuned biomarkers for severe CAP risk stratification. Their high NPV enables safe outpatient management of low-risk cases, while directing intensive resources to high-risk children. Future work should focus on translating these ratios into clinical decision-support tools and exploring their role in guiding immunomodulatory therapies.

## Data Availability

The original contributions presented in the study are included in the article/Supplementary Material, further inquiries can be directed to the corresponding author.
